# Internet of Things and Artificial Intelligence in Healthcare During COVID-19 Pandemic—A South American Perspective

**DOI:** 10.3389/fpubh.2020.600213

**Published:** 2020-12-16

**Authors:** Parag Chatterjee, Andreína Tesis, Leandro J. Cymberknop, Ricardo L. Armentano

**Affiliations:** ^1^Bioengineering Research and Development Group (GIBIO), Universidad Tecnológica Nacional, Buenos Aires, Argentina; ^2^Department of Biological Engineering, Universidad de la República, Montevideo, Uruguay

**Keywords:** internet of things, artificial intelligence, machine learning, healthcare, ubiquitous, virtual healthcare, COVID-19, pandemic

## Abstract

The shudders of the COVID-19 pandemic have projected newer challenges in the healthcare domain across the world. In South American scenario, severe issues and difficulties have been noticed in areas like patient consultations, remote monitoring, medical resources, healthcare personnel etc. This work is aimed at providing a holistic view to the digital healthcare during the times of COVID-19 pandemic in South America. It includes different initiatives like mobile apps, web-platforms and intelligent analyses toward early detection and overall healthcare management. In addition to discussing briefly the key issues toward extensive implementation of eHealth paradigms, this work also sheds light on some key aspects of Artificial Intelligence and the Internet of Things along their potential applications like clinical decision support systems and predictive risk modeling, especially in the direction of combating the emergent challenges due to the COVID-19 pandemic.

## Introduction

Recent years have seen a tremendous surge in domains like the Internet of Things (IoT). The area of healthcare has always been one of the principal application domains of applied IoT. Clubbed with recent advances in Artificial Intelligence (AI) and Machine Learning, eHealth has emerged to new heights in recent times. With the approach of the COVID-19 pandemic, the entire healthcare sector across the world has received shudders from multifarious aspects, including its capacity and deliverability, promptness in response, connected information and analysis. IoT and AI applied to healthcare makes the domain of eHealth largely transdisciplinary, and especially during the times of COVID-19 pandemic, has opened new frontiers of challenges. On one hand, the aspect of eHealth has been fortified in South America during the pandemic by fast development of virtual healthcare solutions. On the other hand, technological areas like IoT and AI have received a strong push to provide fast and efficient healthcare services especially in the perspective of COVID-19, often to automate and facilitate several tasks of the healthcare personnel.

This work is focused on highlighting some of the key aspects of IoT and AI in healthcare related to the domain of South America, especially relevant during the times of COVID-19 pandemic. The principal aspects of eHealth services during COVID-19 in South America have been illustrated, followed by a country-wise review of the state-of-the-art tools and solutions like mobile apps and virtual consultations. Overall, an enormous technological response in healthcare in the South American domain has been noted during the COVID-19 pandemic.

## Key Issues in Healthcare Services During COVID-19 Pandemic

With the advent of COVID-19, the entire healthcare system in many countries across the world faced the tremendous pressure of the newly infected patients. Added to the pressure was the extremely critical protocols (1) of water, sanitation, hygiene, and waste management, maintaining which implied new challenges in handling patients in the hospitals and healthcare centers. However, these challenges during the times of pandemic opened new possibilities for IoT and AI in the domain of healthcare. IoT is basically an interconnection of different objects, mostly heterogenous, to share important data among each other, aiming to provide more efficient services to the users. Artificial Intelligence on the other hand contributes significantly in making sense of the huge data accumulated from various sources by processing it and applying intelligent analytics to get deeper insights, in addition to providing smart services to the stakeholders. Since the inception of the COVID-19 pandemic, the healthcare sector in the entire South America faced several challenges, the principal ones being the shortage of human resources in essential healthcare services, arrangement of treatments for usual patients, and suddenly surged demand of specific medical supplies.

### Healthcare Personnel

The crisis occurred due to the COVID-19 pandemic resulted in a shortage of available medical staff to treat the usual patient population. Due to the shortage, special efforts were taken by several countries to address the sudden surge in the need of physicians in the healthcare system. Several South American countries like Argentina, Uruguay, Chile and Venezuela have formed cooperation between the university faculties of medicine and state healthcare authorities to invite and engage medical students in the combat of COVID-19 on one hand, and summoned retired physicians to join the existing healthcare staff during the pandemic (2–7). For example, in Uruguay, retired physicians were invited for voluntary participation in the follow-up of patients under home-quarantine through tele or videoconference. Also, there has been a movement of physicians between countries to handle the shortage of healthcare staff due to the pandemic's effect on the healthcare system (8).

### Patient Follow-Up

Due to the added risk of contamination and to reduce gatherings in hospitals and healthcare centers, the aspect of remote healthcare especially for the existing non-critical patients stood highly important. Also, to free up healthcare resources for the critical COVID-19 patients, most of the non-critical patients with other diseases were advised to avoid hospital visits (9). Clearly, this involved a disruption in the usual treatment and follow-up of the existing non-COVID patients, leading to newer challenges in maintaining a necessary level of treatment and follow-up on one hand, and keep provisions for COVID-19 patients on the other hand.

### Medical Resource Shortage

Due to non-uniform geographical distribution of the COVID-19 cases, there has been frequent need of sharing and interprovincial transportation of medical supplies among different healthcare facilities in a dynamic manner based on the progression of COVID-19 cases, to avoid shortage at a specific facility with high demand. To avoid the shortage of essential medical devices like respirators and rapid test kits, several collaborations have been established between academia and industry, for the development of devices like artificial respirators or UV-based sterilizing devices (10, 11).

## Principal Applications of IoT and AI in Virtual Healthcare—South American Perspective

The application domain of Artificial Intelligence in healthcare is extremely wide. In addition to intelligent analytics and decision-making, AI and IoT have specific advantages in eHealth during the emergent situations due to COVID-19. Especially in South America, where challenges in healthcare are multifarious like inequalities in healthcare access, healthcare quality, demographic and epidemiological changes in the population (12), the situation due to COVID-19 created a substantial pressure on the healthcare system. But this opened a vast potential for applying eHealth, making the relevance of IoT and AI paramount. Especially in the perspective of COVID-19, several aspects of eHealth using IoT and AI can be applied for improved treatment and monitoring and better management of healthcare services during the pandemic times.

### Virtual Consultations and Remote Monitoring With IoT Devices

To avoid the hospital visits and reduce the conglomeration of non-critical patients in healthcare facilities during COVID-19, several South American countries have started virtual consultation systems through their public healthcare system (Table 1). On one hand, some countries enhanced their existing telehealth platforms, and on the other hand, dedicated virtual consultation channels were opened attributed to the pandemic. It is mostly performed through telephone calls or videoconferencing or even through messages, but without an extensive usage of remote monitoring devices.

**Table 1:** State of virtual consultations during COVID-19 in South America.

**Country**	**State of virtual consultation with physicians during COVID-19**
Argentina	The *Tele-Covid* service was launched in the context of COVID-19, enabling people covered under public healthcare system to perform consultations to medical specialists through video-calling. Apart from supporting the follow-up of people affected by COVID-19, it also extends support to other risk groups like people with chronic diseases, pregnant women, newborns, children under 1 year of age, people with disabilities and those requiring psychological support (13).
Bolivia	A telemedicine initiative *(Telesalud Bolivia)* was undertaken with the aim of reaching the entire population, including call centers, and resources for training for respective stakeholders (14).
Brazil	A strong initiative was taken including the approval of a national law to use telemedicine during COVID-19 and also in the future, dealing with technology-linked healthcare, research, and prevention of diseases. Also, the *RadVid-19*, an artificial intelligence tool to identify different areas estimating the probability of a COVID-19 case was introduced (15, 16).
Chile	A new model of telehealth *Hospital Digital* was established (with upcoming features like access to personal clinical history and EHR), in addition to the initiatives under National Digital Strategy of Healthcare (17).
Colombia	The telemedicine model supported in relieving the pressure of the healthcare system during the pandemic by offering services like teleconsultations and delivery of medicines to more than 30 million people, in addition to existing telehealth initiatives like TeleUCI, for remote intensive care units (18).
Ecuador	Through the telemedicine platform, patients are provided access to specialist physicians virtually (even through Zoom or WhatsApp for the private system). Especially during the pandemic, the call center also complemented the telemedicine platform for general queries (19).
Guyana	Several initiatives have taken since the last few years for universal healthcare and a better outreach. Apart from specific telemedicine missions, websites were designed especially during COVID-19 with personalized chat options for specific support.
Paraguay	The implementation of telemedicine was aimed at in a pre-pandemic scenario to universalize health services. In addition to enabling carrying out different procedures online, a remote monitoring service for supplies and medicines was set up (20). Also applied research was performed on virtual healthcare platforms toward universal healthcare coverage, through diagnostic imaging studies of tomography and ultrasound, and other biological electrical signals like Electrocardiogram (ECG) and electroencephalogram (EEG) and evaluating the mechanism of sharing the information with specialists for diagnosis.
Peru	The telemedicine platform *TeleSalud* is aimed at facilitating virtual consultations with specialists across Skype or Zoom, primarily by establishing link between different stakeholders of the healthcare system (21).
Suriname	Rural telecommunication network expansion and enhancement programs were undertaken previously to increase the reach of services like telehealth. However, currently no substantial instances of virtual consultations were noted.
Uruguay	People with symptoms of COVID-19 have direct access to online healthcare portal, leading to FAQs and other essential support. For non-COVID cases as well, the telehealth system is accessible for virtual consultations with medical specialists, and other usual services like scheduling appointments and access to personal clinical history.
Venezuela	Despite issues with the shortage of resources for telemedicine, the usage of social media for virtual consultations has seen a sharp increase, often used as an alternative medium of communication with the physicians (22).

However, the system of virtual consultation is primarily built on the foundation of verbal reporting of the symptoms by the patient and the physician's diagnoses based on that. A key to efficient diagnosis is the knowledge of different health indicators in addition to the detailed symptoms reported by the patient. But through telephone calls or videoconferencing, this stands as a substantial challenge, as in virtual consultations, the physician has to entirely depend on the reported symptoms. Also, it raises the possibilities of patients lacking confidence in the diagnosis since it is prepared without any precise knowledge of the health indicators.

IoT-eHealth devices hold the potential to play a significant role in this area. It aims at monitoring patients remotely from their homes using smart healthcare devices, to avoid or minimize their hospital visits on one hand, and to provide more accurate health-information to the physicians before or during virtual consultations. IoT-eHealth devices are usually capable of measuring several health parameters like heart rate, blood pressure, temperature, blood glucose etc. right from the patient's home and send summarized information to the medical personnel, to facilitate their diagnosis and treatment. For example, significant surge has been seen in the usage of devices like pulse oximeters in the COVID-19 scenario, to monitor blood oxygen levels (SpO_2_) at home itself. Especially during the times of COVID-19, the usage of smart healthcare devices through IoT platforms is proposed, as it ensures better visibility of the patients' health to the physicians during the virtual consultations and also facilitates more accurate and efficient treatment.

### Early Detection, Diagnosis, and Management Using Artificial Intelligence

Due to the COVID-19 pandemic, almost all the countries in South America faced an enormous load in the healthcare system. This involved scarcity of resources like medical staff, or even space in hospital and healthcare facilities. This opened a strong need to automate the basic decision-making processes for providing advice to the patients (35–38). AI plays an important role in this aspect, especially supporting physicians in early detection of COVID-19 cases by quickly analyzing irregular symptoms and other suspicions and thus alert the respective stakeholders like the patients and healthcare authorities (39).

**Figure 1:**
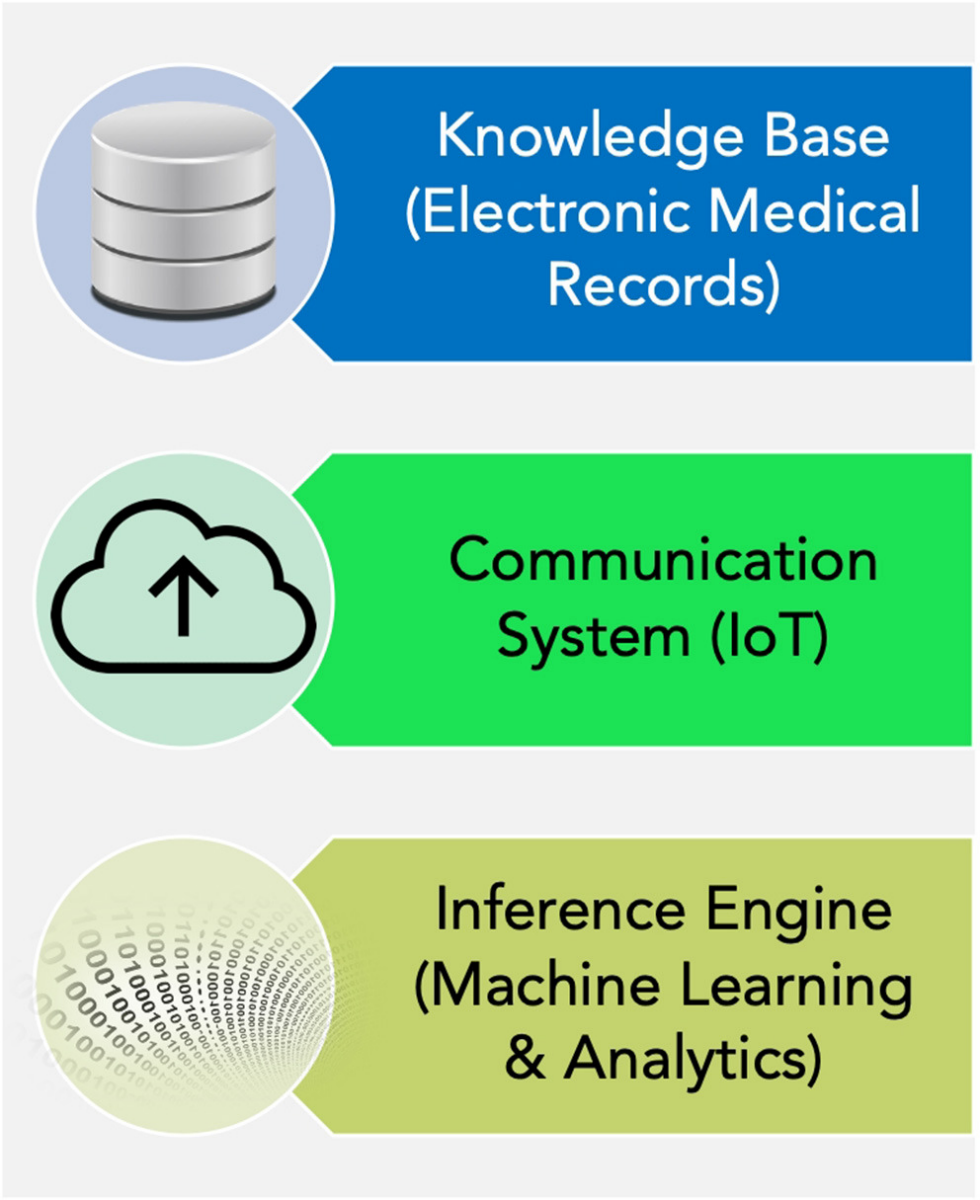
Clinical decision support system.

Almost all the countries in South America have developed mobile applications and web-platforms to analyze the symptoms provided by people and matching it with predesigned decision trees, to provide specific advice related to COVID-19 (Table 2). Using IoT-eHealth devices counts more significant in this domain because of its more precision in reporting health indicators remotely and its potential in facilitating the automated decision-making process through. Since the quality of input health-data to the intelligent models through smart eHealth devices would be better than just self-reporting of symptoms, the advice would be more specific, personalized and efficient. Clinical decision support systems hold the potential to provide fast and automatic primary responses to the patients based on the health-data obtained (Figure 1), saving significant resources like time and personnel in the rush-hours and times of pandemic.

**Table 2:** Mobile and web-apps in South America for early detection of COVID-19.

**Country**	**Mobile Apps during COVID-19**
Argentina	The *Cuidar* mobile app (23) enables self-diagnosis for people based on manual entry of symptoms, provides assistance and recommendations in the case of contagion and provides contact-tools for these cases to health authorities. The application is linked to a broader system that articulates the collected information with the health-authorities in charge of emergency care, both for the national and provincial governments. In this way, the app complements and assists the prevention and care policies for the population and, in particular, provides specific elements and supplies for the health intervention of the Ministries of Health throughout the national territory of Argentina.
Bolivia	The *Coronavirus Bolivia* app includes comprehensive information on symptoms, official communications, notices, information, emergency contacts and FAQs (24).
Brazil	The *Coronavirus-Sus* app was launched with the purpose of alerting people regarding the situation through information on diverse topics like possible symptoms, preventive measures, or detection in case of symptoms. In case of suspected infection, it can be checked if the symptoms are compatible with COVID-19, and if so, instructions are provided, referring to the nearest basic healthcare facility (25).
Chile	The *CoronApp* includes health status reporting, daily monitoring of symptoms, controlling the quarantine, resolving doubts and reporting risk situations to prevent infections, in addition to providing updated information from the health authorities (26). It has a virtual assistant based on artificial intelligence to answer general queries by WhatsApp, or by phone call, contacting the healthcare authorities. In addition, using geolocation of the phone, the app has been enabled to send alerts and notifications to people if they leave their quarantine zone, and reporting of risk situations in the form of crowds, events and improper conduct. The motive is a collaborative care system in connection with the health authorities and administrations to take quick actions in risky situations.
Colombia	The *Coronapp* is a free app that does not consume data and it helps in the detection of affected areas and nearby people with a positive diagnosis for COVID-19. Also, it facilitates the real-time monitoring of data collected at the Emergency Operations Center of the National Health Institute (INS), so that they can act quickly and provide support in coordination with local, departmental and national authorities (27).
Ecuador	The *SaludEc* app is oriented to meet several objectives. It is aimed at evaluating people's symptoms to flag possible cases or rule out the possibilities. It is also a way to send official information timely through digital channels and reduce the load on channel 171. It puts forward a telemedicine channel to complement the already established channels. Also, it enables the scheduling of medical appointments in the first level health centers of the Ministry of Public Health in specialties not related to the coronavirus such as General Medicine, Psychology, Obstetrics and Dentistry (28).
Guyana	With the objective of improving the response of the ministry to reports of suspected cases of COVID-19, the Ministry of Public Health has launched an online app, providing self-screening facilities based on symptoms, in addition to regular recommendations and alerts, and other useful information like hotline numbers and periodic information on COVID-19 (29).
Paraguay	The *COVID-19 PY* platform is aimed exclusively at people who are in isolation and health personnel to connect through the web-app. It offers daily medical self-reporting by people in home-quarantine and if symptoms are detected, corresponding recommendations are provided. Also monitoring and follow-up of people in quarantine is performed using geolocation for greater efficiency in the control of data of people in isolation (30).
Perú	The *Perú en tus manos* app shows a heatmap with red circles in the areas with already infected people and with orange circles where there are people with registered symptoms of COVID-19. Additionally, the app gives people the possibility of carrying out a digital triage oriented to the epidemiological alert to find out if they are at risk of having transmitted the coronavirus. Likewise, an important functionality has been developed for patients affected by COVID-19 under compulsory social isolation, enabling them to report their health status and update their symptoms regularly, so that the members of the '*Te Cuido Peru'* Group in coordination with the Ministry of Health can attend them in a timely way. In this way, they also receive a daily summary and follow-up of their health condition and will know if their health has deteriorated. If necessary, health specialists can contact the affected person to provide immediate care if needed (31).
Suriname	A dedicated website for COVID-19 has been developed, primarily for the dissemination of official information and guidelines related to the disease, in addition to reporting systems integrated with the website (32).
Uruguay	The mobile app *Coronavirus UY* allows to connect citizens with possible symptoms of COVID-19 with the healthcare providers, in order to reduce waiting times for consultations and attention during the state of health emergency in Uruguay, and provide other uses like visualization of information across the country, reporting of symptoms, telemedicine consultation and exposure alerts. Exposure alert is a valuable tool both for people (to be able to quickly find out about a certain risk of contagion) and for the authorities, in terms of monitoring and responding to the appearance of specific sources of contagion. In order to provide total transparency and guarantees on the management of the information collected, in this first stage, the national-level stakeholders (academia, industry, organized civil societies) were given the possibility of auditing the documentation and source code of the Coronavirus UY app, including its exposure alert functionalities (33).
Venezuela	A COVID-19 screening survey through the mobile *Patria* system was launched, enabling people to report the appearance of symptoms related to the disease, facilitating the follow-up by authorities (34).

### Connected Electronic Health Records (EHRs)

The advantage of the electronic health-data acquired by the IoT-eHealth devices lies in its transferability and processability. The IoT offers a sharing platform so that the EHR collected from the patients' eHealth devices can be shared across multiple heterogeneous devices, and across health networks in an easily accessible way to the healthcare providers. In South America, several countries have developed models for shareable EHR, enabling seamless exchange of EHR between healthcare providers as well as administrative entities. Primarily it deals with electronic versions of medical reports, physician's prescriptions and other information of patients stored online, making it easy to access for the patients avoiding visits to healthcare facilities and also for the healthcare personnel to provide remote treatment more efficiently. With the advent of COVID-19, the need of sharing health-data has increased significantly, in order to share important aspects of the disease outbreaks, compare symptoms across different regions and healthcare facilities and to understand the comprehensive scenario. IoT supports this scenario through multilayered sharing of EHR among different stakeholders, to provide comprehensive support to the patients.

Especially during the times of COVID-19, shareable and comprehensively accessible EHR enables to design a detailed risk-profiling of the population in almost real-time, using clustering algorithms. Also, it helps in deciphering the health-trend of the population mostly through supervised predictive models, strongly important in day-to-day planning and decisions on healthcare services during the pandemic.

### Priority Scheduling of Patients

After the first effect of COVID-19 pandemic, as several healthcare facilities started opening for usual healthcare services in different South American countries, one of the key challenges is to manage the huge list of waiting patients who had to avoid visits the hospitals during the times of lockdown. The handling of patient-queues and priority lists is important, as delays in patients with potential risk could be fatal and due to limited resources of healthcare facilities, prioritization should be done efficiently and accurately. AI can process the comprehensive health-data of patients, including the previous hospital visits, laboratory results and interim treatments or complications, and use machine learning to train models and generate priority lists of patients to facilitate the medical staff in attending usual patients after lockdown.

### Resource Sharing and Policymaking

AI plays a significant role in resource optimization. Especially in the crisis of resources and medical supplies in the times of COVID-19 pandemic, applying unsupervised learning techniques like clustering is useful to identify the key clusters and overall situation of resources across different centers, followed by distribution algorithms to share the resources efficiently. It is useful in different aspects of policymaking in emergency situations. Also, it can be used to predict the shortage of resources and supplies with considerable anticipation, giving enough time to organize the transfer and management of resources in combating the shortage.

## Conclusion

The domain of healthcare has been one of the fastest adopting sectors for IoT and AI (40–47). Especially in the times of COVID-19 pandemic, the entire healthcare sector faced new challenges. In this context, several opportunities for application of IoT and AI have been discussed pertaining to a South American perspective. It includes efficient virtual consultations and remote monitoring of patients, intelligent diagnoses, sharing EHRs, and priority scheduling for patients. Several countries in South America face challenges in areas like digital-divide and disparity in population having access to digital technologies toward healthcare. Apart from that, despite having other challenges like limited power in handling big data, interoperability of health-data among heterogeneous stakeholders, and lack of unified implementational structure for eHealth, AI and IoT put forward immense potential in the healthcare sector, especially during and after the COVID-19 pandemic.

## Author Contributions

All authors listed have made a substantial, direct and intellectual contribution to the work, and approved it for publication.

## Conflict of Interest

The authors declare that the research was conducted in the absence of any commercial or financial relationships that could be construed as a potential conflict of interest.
